# Extirpation of a Part of a Rib for Neuralgia

**Published:** 1840-01

**Authors:** E. H. Dixon


*Extirpation of a part of a Rib for Neuralgia.* By E. H. Dixon.

Oct. 16, 1838.—Jane Bailey, aged thirty, two years ago, was overturned in a carriage, and dragged something like a mile, receiving various contusions, and being taken out insensible. No lasting injury was the result, with the exception of a severe pain over the tenth rib of the left side, for which she received no treatment at the time, saving the customary bleeding practiced on such occasions. A few weeks after the injury, the affected spot became the seat of a small irregular projection, and of exquisite and constant pain. This pain I conceive to have been neuralgic in character, from the commencement, inasmuch as it was occasionally of equal violence for weeks together, and not accompanied with other signs of inflammation. It would subside partially for a few hours and then recommence with such intolerable severity, that the patient assured me death was anticipated as a relief, to her misery. She had some little cough, with no expectoration; the cough I concluded to be the result of irritation, and constant loss of sleep, as I could detect no signs of pulmonary disease. The pain extended forwards and upwards, (from the projection which seemed to be near the sternal extremity of the rib,) over the stomach; upon turning her head and shoulders to the opposite side, so as to throw out the ribs, the projection was evidently caused by the end of the rib, as though the cartilage had been broken off. I at first thought it a fracture, with a redundant growth of bone, but, upon further examination, concluded the rib to be natural, though I could not account for its projection, without supposing a fracture to have occurred between the prominence and the vertebral articulation, thus destroying the natural curvature of the rib, opposing the action of the intercostal muscles, and favoring the absence of the cartilage; all this would sufficiently account for the projection. During the two years preceding the operation, the patient had been repeatedly cupped over the affected region, with momentary relief; part of this time was spent in the New York Hospital; her pain over the stomach being very acute; she had been treated whilst there for organic affection of that viscus. This, however, it seemed rational to explain, upon the principle of pain being continued from the irritated nerve, to its distribution; the anterior branch of the intercostal nerve going to the muscles and integument over the stomach. It was evident that counter irritation, however severe, would produce no benefit in such a case, and as the extirpation of the part of the nerve itself was impossible, without the certainty of opening the cavity of the pleura, I resolved to remove the projecting portion of the rib, hoping that when the tension was removed, the pain would cease.

I removed about two inches of the rib, by means of a very cautious dissection; leaving the cavity of the pleura unopened; the cartilage was not attached, being, most probably, absorbed. There was nothing peculiar or difficult in the operation; not a bad symptom ensued; the wound healed by adhesion in a week, the pain was removed instantly, and has not returned; the patient has gained twenty-three pounds of flesh, a sufficient evidence that a severely irritating cause had been removed.—*New York Journal of Medicine and Surgery.*

